# Hallmark microRNA signature in liquid biopsy identifies hepatocellular carcinoma and differentiates it from liver metastasis

**DOI:** 10.7150/jca.59933

**Published:** 2021-06-01

**Authors:** Victor Chun-Lam Wong, Ming-In Wong, Chi-Tat Lam, Maria Li Lung, Ka-On Lam, Victor Ho-Fun Lee

**Affiliations:** 1 OncoSeek Limited, Hong Kong Science and Technology Parks, Hong Kong Special Administrative Region, People's Republic of China.; 2 Department of Clinical Oncology, LKS Faculty of Medicine, The Hong Kong Special Administrative Region, People's Republic of China.

**Keywords:** Hepatocellular carcinoma, miRNA signature, Liquid biopsy, Machine learning, HCC diagnosis, HCC screening

## Abstract

**Purpose:** This study aims to develop a liquid biopsy assay to identify HCC and differentially diagnose hepatocellular carcinoma (HCC) from colorectal carcinoma (CRC) liver metastasis. **Methods:** Thirty-two microRNAs (“HallMark-32” panel) were designed to target the ten cancer hallmarks in HCC. Quantitative PCR and supervised machine learning models were applied to develop an HCC-specific diagnostic model. One hundred thirty-three plasma samples from intermediate-stage HCC patients, colorectal cancer (CRC) patients with liver metastasis, and healthy individuals were examined. **Results:** Six differentially expressed microRNAs (“Signature-Six” panel) were identified after comparing HCC and healthy individuals. The microRNA miR-221-3p, miR-223-3p, miR-26a-5p, and miR-30c-5p were significantly down-regulated in the plasma of HCC samples, while miR-365a-3p and miR-423-3p were significantly up-regulated. Machine learning models combined with HallMark-32 and Signature-Six panels demonstrated promising performance with an AUC of 0.85-0.96 (*p* ≤ 0.018) and 0.84-0.93 (*p* ≤ 0.021), respectively. Further modeling improvement by adjusting sample quality variation in the HallMark-32 panel boosted the accuracy to 95% ± 0.01 and AUC to 0.991 (95% CI 0.96-1, *p* = 0.001), respectively. Even in alpha fetoprotein (AFP)-negative (< 20ng/mL) HCC samples, HallMark-32 still achieved 100% sensitivity in identifying HCC. The Cancer Genome Atlas (TCGA, n=372) analysis demonstrated a significant association between HallMark-32 and HCC patient survival. **Conclusion:** To the best of our knowledge, this is the first report to utilize circulating miRNAs and machine learning to differentiate HCC from CRC liver metastasis. In this setting, HallMark-32 and Signature-Six are promising non-invasive tests for HCC differential diagnosis and distinguishing HCC from healthy individuals.

## 1. Introduction

Hepatocellular carcinoma (HCC) is a highly fatal cancer with a death toll over 810,000 deaths every year worldwide 1. Most HCC patients are detected only in advanced stages with a dismal prognosis of less than one-year overall survival 2-4. Diagnostic and treatment delays are often linked to worse survival outcomes in HCC 5, 6. Nearly 20% of patients wait for more than three months from clinical presentation to diagnosis, which is close to the tumor volume doubling time of HCC 6, 7. Therefore, early diagnosis is the pivotal key to improving outcomes for HCC. Over the last decade, the advent of biotechnology has been expediting liquid biopsy application in various clinical settings.

Developing a liquid biopsy assay for HCC diagnosis is clinically useful for several reasons. First, liquid biopsy could potentially complement conventional tissue biopsy for HCC diagnosis. Tissue biopsy has common pitfalls including sampling bias and inaccessibility for safe biopsy. For instance, invasive biopsy procedures are sometimes not feasible for HCC patients with tumors located adjacent to major blood vessels or patients with coagulopathy due to imposing high intra-abdominal bleeding risk 8, 9. In contrast, liquid biopsy technology is non-invasive, repeatable and thus can provide a safe and timely diagnosis of early HCC.

Second, liquid biopsy could potentially complement the detection limit in the traditional approaches for non-invasive HCC surveillance and diagnosis. For screening early-stage HCC, liver ultrasound combined with AFP testing could only achieve 63% sensitivity 10. The sensitivity could be further reduced by 19 - 56% in obesity and chronic liver disease conditions 11. Computed tomography (CT) and Magnetic Resonance Imaging (MRI) sensitivities for 1-2 cm HCC are 65% and 80%-92%, respectively, but the sensitivities plummet to 10% and 34%-71% for early HCC tumors <1 cm 12. An additional liquid biopsy technology may improve the performances of current HCC surveillance strategy.

Third, liquid biopsy could aid the differential diagnosis of liver nodules, especially those with equivocal imaging features and those not accessible to biopsy. Since the liver is a common site for metastasis, differentiating HCC from liver metastasis patients is essential for treatment guidance. For instance, gastrointestinal cancers are known to have a high tendency to metastasize to the liver through the portal vein. Furthermore, patients with underlying chronic liver diseases may have a higher risk of HCC and CRC 13, making the differential diagnosis more essential. More confusingly, a subset of CRC is indistinguishable from HCC with respect to the serological AFP and carcinoembryonic antigen (CEA) levels. Approximately 2.6% (5/193) of CRC patients are positive in the AFP test 14 and 45% (9/20) of AFP-positive CRC patients are negative in the CEA test 15. Therefore, it is an unmet need to develop a liquid biopsy test to differentiate HCC from colorectal cancer liver metastasis (CRCLM).

This study aims to develop a non-invasive liquid biopsy microRNA (miRNA) assay for HCC diagnosis. We began by selecting 32 miRNAs (“HallMark-32” panel) known to regulate the ten hallmarks in HCC. Subsequently, we identified six signature miRNAs (“Signature-Six” panel) based on their expression profiles. HCC-specific diagnostic models were then developed by supervised machine learning. The model diagnostic performances were evaluated using 133 plasma samples from HCC, CRCLM, and healthy individuals. The goal of this study is to develop a reliable liquid biopsy for HCC identification and differential diagnosis.

## 2. Methodology

### 2.1. Patient & blood collection

Patients diagnosed with HCC and CRCLM from July 2015 to Dec 2019 at Queen Mary Hospital were recruited. All HCC samples were from patients diagnosed with Barcelona Clinic Liver Cancer (BCLC) intermediate stage B. All CRCLM samples were from patients diagnosed with CRC having synchronous liver metastasis. All blood samples were obtained after written informed consent. A total of 133 samples were collected including 22 healthy individuals, 63 CRCLM, and 48 HCC patients. Eight milliliters of peripheral blood samples were collected in the Streck Cell-Free DNA tubes (Streck Inc., USA). The study was approved by the Institutional Review Board of the University of Hong Kong.

### 2.2. Sample preparation and miRNA isolation

Blood sample were spun down at 2,000g for 20 minutes at 18°C, followed by 16,000rpm centrifugation for 10 minutes at 10°C. The plasma was then transferred to a new 1.5mL centrifuge tube and stored at -20°C. After centrifugation, the supernatant was further precipitated by isopropanol, followed by RNA purification using a column-based silica membrane technology (MACHEREY-NAGEL, Germany). The eluted RNA was subjected to PolyA tailing reaction with PolyA polymerase and ATP and incubated for 60 minutes at 37°C, followed by 5-minute deactivation at 70°C. Subsequently, cDNA synthesis was performed by adding oligo-dT adapter primer, miRNA-specific forward primer (Table S1), reverse transcriptase and then the reaction was incubated for 20 minutes at 42°C, followed by 5 minutes at 85°C.

### 2.3. miRNA analysis

Roche LightCycler 480 was used for qPCR reactions. The CT values for microRNA targets were generated by the second derivative maximum of the fluorescence curve. For the miRNAs that have no CT value after calculation, a CT value of 45 was filled to indicate low expression level for the subsequent diagnostic modeling, and the CT values were converted to fold differences by the equation 2^(-ΔCt). Samples missing more than 50% of miRNA CT values were excluded from this study. Analysis of variance (ANOVA) and Student's T-test was applied to examine the difference of miRNA expression between HCC, healthy, and CRCLM groups using the statistic package in python. The p-values <0.05 were considered statistically significant. The seaborn and matplotlib package in python were used for results visualization.

### 2.4. Data preprocessing and machine learning models

Data were split into training (n=106) and test (n=27) sets with an 80% splitting ratio. Samples were balanced by using the imblearn package and Scikit-learn library in the python environment. Four supervised classifier algorithms: Artificial Neural Network, Random Forest, Gradient Boosting Classifier, and Logistic Regression were tested for their performances to distinguish HCC samples from healthy and CRCLM samples. The diagnostic model prediction outcome is binary; HCC samples were assigned as one, while CRCLM and healthy individuals were assigned as zero. For model improvement, the sample quality data included RNA concentration and their 260/280 and 260/230 absorbance ratios, cDNA concentration and their 260/280 and 260/230 absorbance ratios, and the specificity of each miRNA melting curve. All features were standardized by the StandardScaler package in python. For each algorithm, 30 models were first developed, and their mean accuracy was taken for evaluation. To further examine the performance of the algorithms, sensitivity, specificity, positive predictive value (PPV), negative predictive value (NPV), area under curve (AUC), receiver-operating-characteristic (ROC) curves were computed in python. All experiments, analysis, and machine learning modelling for the healthy, HCC, and CRCLM were repeated at least three times.

### 2.5. Establishing HallMark-32 panel

To select the miRNA candidates for HCC detection, we reviewed literature that reported miRNA in HCC and then selected miRNAs accordingly to ten hallmark properties proposed by Hanahan and Weinberg 16. The hallmarks included sustaining proliferation signaling, evading growth suppressor, avoiding immune destruction, enabling replicative immortality, tumor-promoting inflammation, activating invasion and metastasis, inducing angiogenesis, genome instability, resisting cell death, and deregulating cellular energetics. All hallmarks of HCC were covered by at least one of the miRNAs in the HallMark-32 panel (Table 1) (Table S2).

### 2.6. TCGA miRNA expression and survival data analysis

The liver hepatocellular carcinoma (LIHC) dataset (version 2016-01-28, n=372) from TCGA database was downloaded. The vital status (dead vs. alive), survival days (days to death or days to last follow-up), and miRNA expressions (reads per million miRNA mapped) were analyzed. Kaplan-Meier (KM) survival curve analysis was performed in SPSS software. Cox proportional hazards analysis was computed in python.

## 3. Results

### 3.1. Discovery of HCC-specific Signature-Six miRNA panel

**Figure 1:**
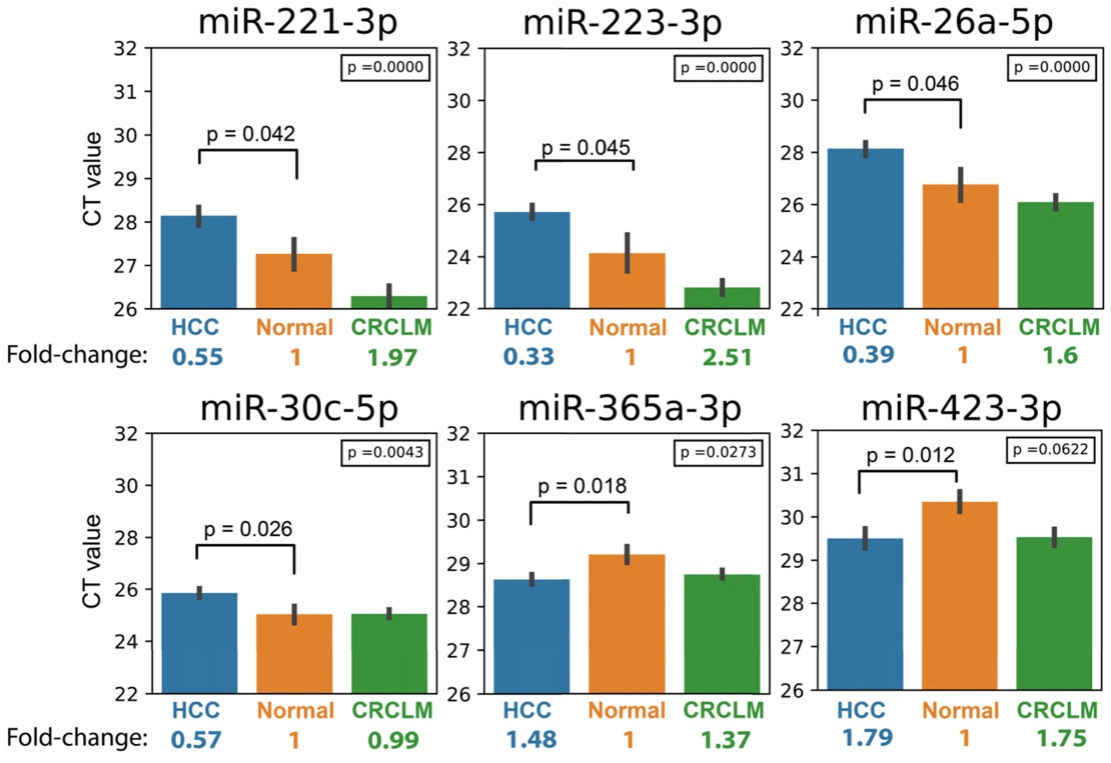
Bar chart showing the expression of Signature-Six in HCC, Healthy and CRCLM specimens, and their fold change relative to healthy. The *p*-values at the upper right corner represent *p*-value in ANOVA test comparing the CT values in three groups (HCC, Healthy and CRCLM). The *p*-values at the top of the bars represent *p*-value in *t*-test comparing HCC and healthy individuals.

To identify signature miRNAs that express differently between HCC and healthy individuals for HCC detection, we examined the expression profiles of 32 miRNAs by qPCR in 70 plasma samples (48 HCC and 22 healthy individuals) (Table S3 and Figure S1). Six signature miRNAs (miR-221-3p, miR-223-3p, miR-26a-5p, miR-30c-5p, miR-365a-3p and miR-423-3p) were identified. The level of miR-365a-3p and miR-423-3p was significantly elevated in the plasma of HCC samples compared to healthy samples, while miR-221-3p, miR-223-3p, miR-26a-5p, and miR-30c-5p were significantly down-regulated (Table 2 and Figure 1). These miRNAs were then consolidated to be the “Signature-Six” panel.

Next, we investigated whether Signature-Six expressed differently among three groups of specimens (HCC, CRCLM, and Healthy) using ANOVA analysis. Five miRNAs in Signature-Six consistently showed statistical significance (Table 2). The expression of miR-223-3p in the Signature-Six panel achieved the highest significance (*p*-value=8.56 x 10^-9^). Furthermore, when comparing the miRNA expressions between HCC and CRCLM, four miRNAs in Signature-Six were significantly different (Table 2). As expected, the internal control miR-451a exhibited no difference in all statistical tests (Table S3). Taken together, the differential expression profile of miRNAs provided a strong foundation for building the HCC diagnostic model.

### 3.2. HCC Diagnostic Model development with Signature-Six and HallMark-32

**Figure 2:**
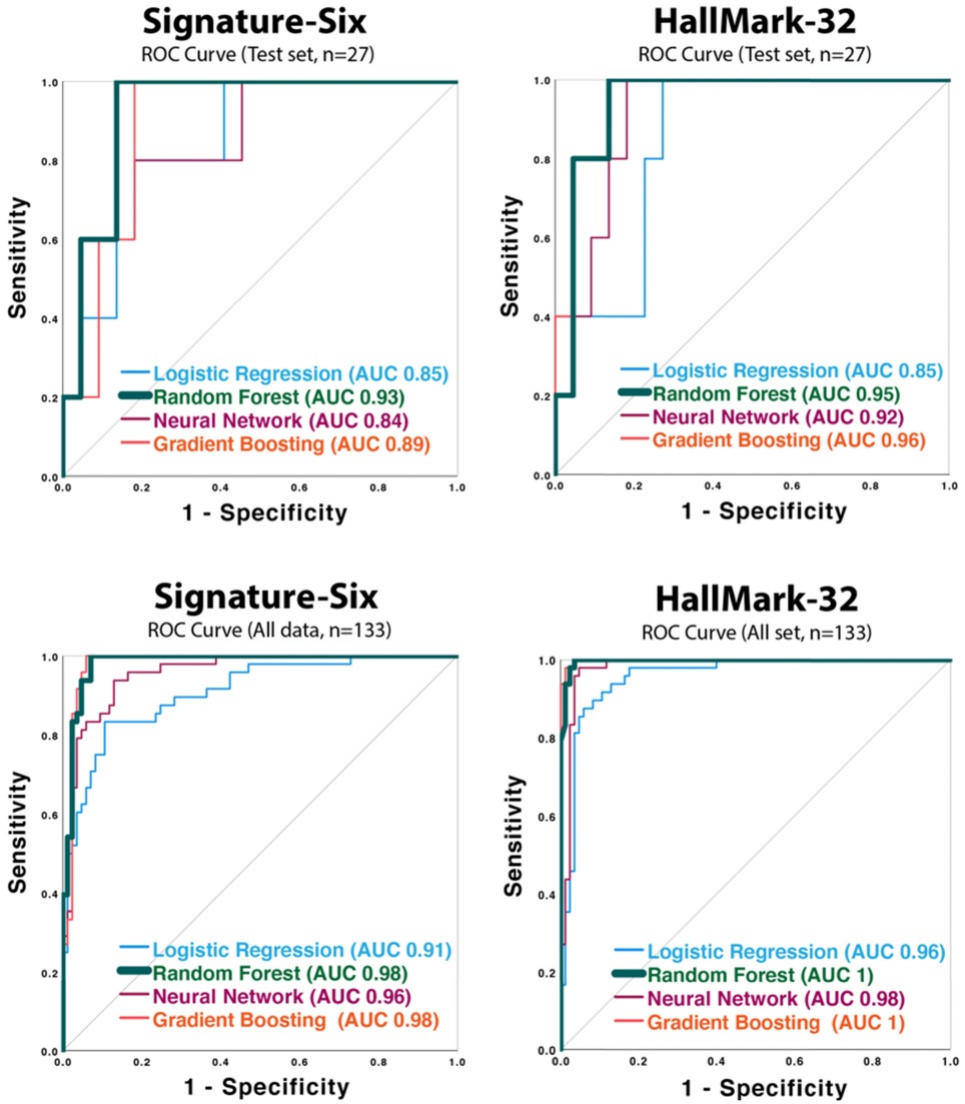
Receiver operating characteristic (ROC) curves of the neural network, random forest, gradient boost classifier, and logistic regression models applied to Signature-Six and HallMark-32 panel in test set (n=27) and the whole dataset (n=133).

To develop an HCC diagnostic model, four supervised machine learning classification models (Neural Network, Random Forest, Gradient Boosting Classifier, and Logistic Regression) were applied. One hundred thirty-three plasma samples (48 HCC, 63 CRCLM, 22 healthy individuals) were utilized. Each machine learning model was developed using a training dataset (n=106), and then the performance was validated in a test dataset (n=27). Performance indices such as accuracy, AUC score, *p*-value for AUC, sensitivity, specificity, PPV, and NPV were evaluated for each model and are summarized in Table 3. ROC curves are shown in Figure 2. All the AUC scores in both HallMark-32 and Signature-Six are statistically significant (*p* ≤ 0.021) (Table 3 and Figure 2). In HallMark-32, the average AUCs from the four algorithms in the test set (n=27) and the whole dataset (n=133) were 0.92 and 0.99 (Table 3A), which were slightly higher than that in Signature-Six (Table 3B, average AUC is 0.88 and 0.96). In both HallMark-32 and Signature-Six, the Random Forest model outperformed other machine learning models based on mean accuracy. In HallMark-32, the Random Forest model provided 91% accuracy compared to 86%, 89%, 84% accuracy in Neural Network, Gradient Boosting Classifier, and Logistic Regression models, respectively (Table 3). The Signature-Six Random Forest model exhibited an AUC score of 0.93 (95% CI: 0.83-1, *p*-value = 0.003), 100% sensitivity, and 86% specificity; the HallMark-32 Random Forest model exhibited an AUC score of 0.95 (95% CI: 0.86-1, *p*-value = 0.002), 91% sensitivity, and 91% specificity. Taken together, the HallMark-32 Random Forest model provided the best performance for proceeding further model improvement.

### 3.3. Model improvement by sample quality adjustment

**Figure 3:**
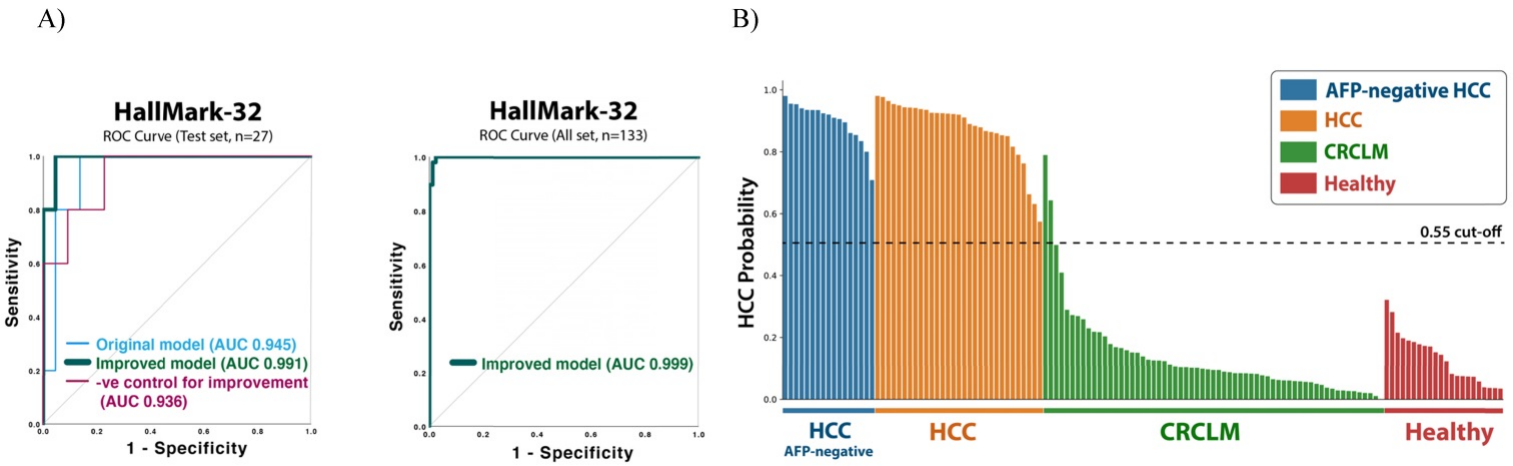
ROC curves of the improved HallMark-32 random forest model and the predictive HCC probability for each sample. A) ROC curves for the test set (n=27) and the whole dataset (n=133). B) The HCC probability predicted by the improved HallMark-32 random forest model (n=133).

Sample quality variation may influence the miRNA detection and, thus, the result of the diagnostic model. To further improve the HallMark-32 HCC diagnostic model, we integrated the sample quality variation into the models. The sample quality features involved 38 parameters covering RNA concentration, RNA 260/280 and 260/230 absorbance ratios, cDNA concentration, cDNA 260/280 and 260/230 absorbance ratios, and the specificity of melting curves (Table S4). After combining these sample quality parameters and the miRNA expression levels, the improved HallMark-32 model contained 70 features in total. After model improvement, the accuracy and AUC of the HallMark-32 Random forest model were increased from 91% to 95% and from 0.945 (95% CI 0.86-1) to 0.991 (95% CI 0.96-1), respectively (Table 4B and Figure 3A). The AUC score of the improved model is more significant than the model before improvement, with a *p*-value of 0.001 and 0.002, respectively. To prove that the success of model improvement was not a random chance, we generated a negative control for the improvement strategy, by replacing the sample quality features with random numbers. As expected, the negative control did not improve accuracy, but conversely, reduced the accuracy from 95% to 84% (Table 4B). The result demonstrated that the integration of sample quality parameters is instrumental and specific for the HCC identification. In summary, with a 0.55 probability cut-off, the test set validation (n=27) demonstrated an AUC value of 0.991 (95% CI 0.96-1, *p* = 0.001), 100% sensitivity, 91% specificity, 0.92 PPV, 1 NPV, and 95% accuracy. By applying the whole dataset (n=133; Table 4A and Figure 3A) to the improved HallMark-32 model, the HCC diagnostic model demonstrated an AUC value of 0.999 (95% CI 0.99-1, *p*=0.000), 100% sensitivity, 98% specificity, 0.96 PPV, and 1 NPV.

### 3.4. Identifying HCC in alpha-fetoprotein-negative samples

Although AFP has been widely used for facilitating HCC screening and diagnosis, the sensitivity only ranged from 41% to 65% 14. Therefore, we interrogated whether HallMark-32 could identify HCC in AFP-negative (< 20ng/mL) samples. To test this, we examined the predicted HCC probability in the AFP-negative samples available in our HCC dataset (n = 17; Figure 3B). The AFP-negative HCC samples were predicted with a high HCC probability (0.76 - 1) by the HallMark-32 model. The improved HallMark-32 Random forest model could identify HCC in AFP-negative samples with 100% sensitivity with a 0.55 probability cut-off (Figure 3B). Collectively, the HallMark-32 model can also identify HCC in AFP-negative samples, suggesting a potential application of HallMark-32 for HCC identification irrespective of serum AFP level.

### 3.5. Clinical association between HallMark-32 panel and patient survival

**Figure 4:**
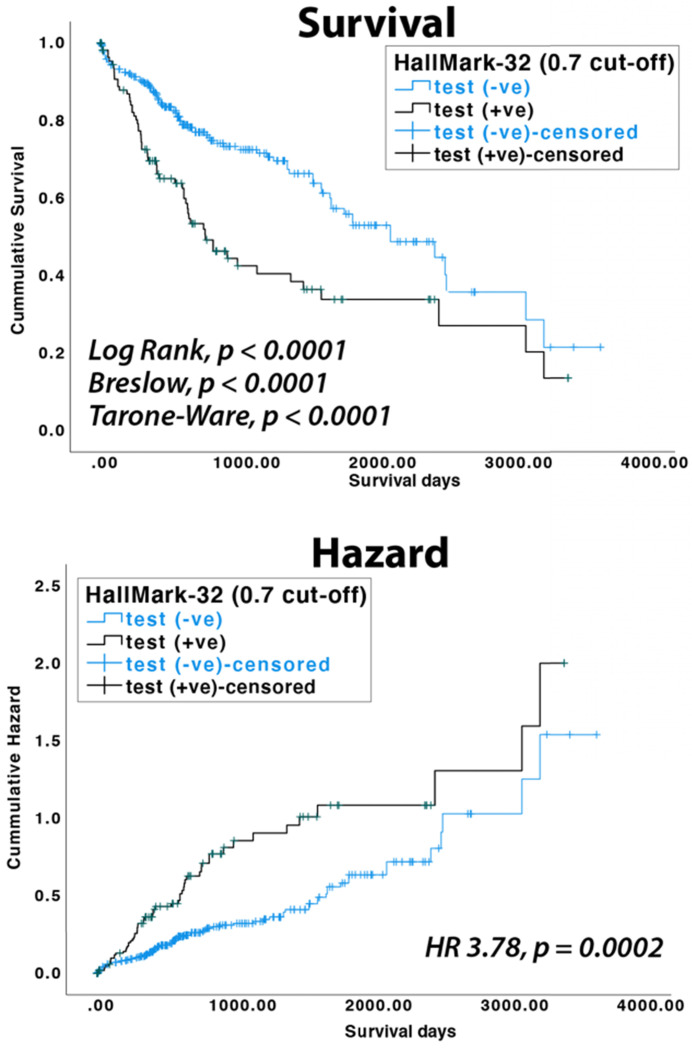
KM survival and Cox proportional hazards analyses of TCGA LIHC dataset (n=372).

To test if the HallMark-32 panel is associated with HCC patient survival, we analyzed miRNA expression data available in the TCGA database. In the TCGA LIHC dataset, 100, 90, 90, and 10 HCC patients belonged to the pathological stages I, II, III, and IV, respectively. Using random forest and a probability cut-off of 0.55, HCC patients with positive prediction results showed a lower survival rate (*p*<0.004 in log-rank test). Using a probability cut-off of 0.7, the clinical association with survival rate is more significant (*p*<0.0001 in log-rank, Breslow, and Tarone-Ware test, Figure 4). HCC patients with positive results have a significantly higher risk of death than patients with negative results (HR = 3.78, 95% CI = 1.70-5.43, *p* = 0.0002 in Cox proportional hazards test, Figure 4). The median survival days for HCC patients with positive and negative results are 770 days (SE 133.7 days) and 2131 days (SE 314.8 days), respectively.

## 4. Discussion

In summary, this study highlighted five key findings: 1) Six signature miRNAs (miR-221-3p, miR-223-3p, miR-26a-5p, miR-30c-5p, miR-365a-3p, and miR-423-3p) were identified. Their expression profiles differed significantly in HCC and healthy individuals, providing a good diagnostic model for non-invasive HCC identification. 2) We reported a strategy to optimize the miRNA-based diagnostic model performance by adjusting sample quality variation. 3) The improved HallMark-32 random forest model demonstrated outstanding performance for identifying HCC, as well as differentiating HCC from CRCLM and healthy individuals. 4) HallMark-32 allowed HCC identification even in AFP-negative samples. 5) TCGA analysis illustrated the clinical association between the HallMark-32 panel and HCC patient survival.

Virtually all cancer progresses with ten cancer hallmarks proposed by Hanahan and Weinberg 16. The development of HCC in hepatitis B virus (HBV)-infected patients, for example, involves dysregulation in p53, PI3K, TGF-β, IL-6, VEGF, and TERT pathways 17, 18, thereby acquiring oncogenic hallmarks for HCC development (Table S5). To develop an HCC-specific assay, we selected miRNA candidates that regulate ten hallmark properties in HCC based on the literature (Table S2). In the expression profiling comparing HCC and healthy individuals, six signature miRNAs (miR-221-3p, miR-223-3p, miR-26a-5p, miR-30c-5p, miR-365a-3p, and miR-423-3p) were identified. Intriguingly, the expression levels of miR-221-3p, miR-223-3p, and miR-26a-5p were significantly lower in HCC and higher in CRCLM than in healthy individuals (Figure 1). The differences of this expression pattern may in part be explained by the cancer stage in HCC and CRCLM patients. For the intermediate-stage of HCC, cancer cells tend to progress by activating angiogenesis and extravasation. Whereas in CRCLM, the extravasation, intravasation, and colonization signaling pathways are activated to develop distant organ metastasis. The differences of the activated signaling pathways may explain the distinct expression patterns. In fact, miR-221-3p, miR-223-3p, and miR-26a-5p are functionally linked to proliferation, invasion, and metastasis in both HCC 19-23 and CRC 24-26. The miR-221 is known to regulate epithelial-mesenchymal transition (EMT) by regulating zinc finger E-box binding homeobox 2 (ZEB2) pathway in HCC. Both miR-223 and miR-26a are linked to invasion and metastasis in HCC. Whereas, in CRC, miR-223 and miR-26a promote proliferation and cell invasion. The miR-221 enhances the cancer stem cell property in CRC by targeting human Quaking (QKI) gene. The signaling interaction between these miRNAs warrants further investigation, but their differential expression pattern could provide an important fingerprint to distinguish primary liver cancer against secondary liver metastases, as demonstrated in this study.

When diagnosing HCC, tissue biopsy procedures are needed, especially when CT and MRI fail to show typical HCC features. However, tissue biopsy may lead to clinical complications and sampling bias. Liquid biopsy may serve as a non-invasive and complementary approach to improve the HCC diagnosis and surveillance. In this study we demonstrated the performance of Signature-Six and HallMark-32 miRNA panel to identify HCC and differentiate HCC from liver metastasis. We also illustrated the capability of HallMark-32 to complement AFP for HCC detection. Taken together, we demonstrated potential applications of Signature-Six and HallMark-32 in supporting HCC surveillance and diagnosis.

Future work with an increased sample size of HCC is needed to evaluate the performance of these models to predict HCC at different stages. Our group has previously conducted technical validation and established clinical utilities of circulating tumor cells in colorectal, lung, breast, gastric, liver, prostate, esophageal, and nasopharyngeal cancers 27-29. The clinical application of combining miRNA, circulating tumor cell 27-29, and bioinformatics technology 30, 31 merits further exploration. To sum up, we discovered circulating microRNA signatures for identifying HCC and demonstrated the promising performance of the liquid biopsy assay for HCC identification and differential diagnosis.

## Supplementary Material

Supplementary figure and tables.

## Figures and Tables

**Table 1:** HallMark-32 panel design based on the published miRNA HCC studies.

HCC Hallmarks	MicroRNA Candidates [References are shown in Supplementary Table 3]
**Sustaining Proliferative Signaling**	150-5p, 125b-5p, 101-3p, 1246, 21-5p, 145-5p, 214-3p, 320d, 18a-5p, 26a-5p, 193a-5p, 19a-3p, 222-3p, 486-5p, 223-3p, 374a-5p, 424-5p, 122-5p, 29a-3p, 451a
**Evading Growth Suppressors**	574-3p, 125b-5p, 23a-3p, 145-5p, 214-3p, 423-3p, 423-5p, 424-5p
**Avoiding Immune Destruction**	23a-3p, 423-5p, 424-5p
**Enabling Replicative Immortality**	1246, 21-5p, 192-5p, 148a-3p
**Tumor Promoting Inflammation**	148a-3p, 30c-5p
**Activating Invasion And Metastasis**	150-5p, 125b-5p, 191-5p, 101-3p, 1246, 21-5p, 145-5p, 125a-5p, 214-3p, 26a-5p, 19a-3p, 148a-3p, 486-5p, 374a-5p, 221-3p, 424-5p, 122-5p, 29a-3p, 451a
**Inducing Angiogenesis**	148a-3p, 423-3p, 424-5p
**Genome Instability And Mutation**	374a-5p, 221-3p
**Resisting Cell Death**	125b-5p, 101-3p, 1246, 145-5p, 125a-5p, 192-5p, 18a-5p, 26a-5p, 193a-5p, 222-3p, 223-3p, 423-5p
**Deregulating Cellular Energetics**	101-3p, 22-5p

**Table 2:** Expression level of miRNAs in Signature-Six, fold-change relative to healthy, and the *p*-value results in *t*-test and ANOVA analyses.

miRNA	Mean (CT values)			SEM (CT values)			Fold-Change relative to Healthy			HCC vs. CRCLM (*p*-value)	HCC vs. Healthy (*p*-value)	HCC vs. CRCLM vs. Healthy (*p*-value)
	HCC	Healthy	CRCLM	HCC	Healthy	CRCLM	HCC	Healthy	CRCLM			
**221-3p**	28.15	27.27	26.29	0.21	0.36	0.25	0.55	1	1.97	0.000**	0.042*	0.000**
**223-3p**	25.72	24.14	22.81	0.25	0.73	0.28	0.33	1	2.51	0.000**	0.045*	0.000**
**26a-5p**	28.14	26.77	26.1	0.26	0.62	0.25	0.39	1	1.6	0.000**	0.046*	0.000**
**30c-5p**	25.85	25.04	25.05	0.17	0.31	0.17	0.57	1	0.99	0.001**	0.026*	0.004**
**365a-3p**	28.63	29.2	28.75	0.12	0.2	0.09	1.48	1	1.37	0.474	0.018*	0.027*
**423-3p**	29.51	30.35	29.54	0.23	0.23	0.19	1.79	1	1.75	0.917	0.012*	0.062

Notes: * *p*-value ≤ 0.05; ** *p*-value ≤ 0.01; High CT value means low miRNA expression.

**Table 3:** Performance of four machine learning algorithms in HallMark-32 and Signature-Six assays.

**A)**	**HallMark-32**												
	**Test set (n = 27)**							**All data (n=133)**					
**Model**	**Mean** **Accuracy ± SD**	**AUC** **(95% CI)**	***p*-value** **for AUC**	**Sensitivity**	**Specificity**	**PPV**	**NPV**	**AUC** **(95% CI)**	***p*-value** **for AUC**	**Sensitivity**	**Specificity**	**PPV**	**NPV**
**Neural Network**	0.86 ± 0.04	0.92 (0.811-1)	0.004	1	0.86	0.88	1	0.98 (0.96-1)	0.000	0.96	0.95	0.92	0.98
**Random Forest**	0.91 ± 0	0.95 (0.86-1)	0.002	0.91	0.91	0.91	0.91	1 (0.99-1)	0.000	0.98	0.98	0.96	0.99
**Gradient Boosting**	0.89 ± 0	0.96 (0.88-1)	0.002	0.91	0.86	0.87	0.9	1 (0.996-1)	0.000	0.98	0.96	0.94	0.99
**Logistic Regression**	0.84 ± 0	0.85 (0.69-1)	0.018	0.91	0.77	0.8	0.89	0.96 (0.93-1)	0.000	0.92	0.88	0.81	0.95
**B)**	**Signature-Six**												
	**Test set (n = 27)**							**All data (n=133)**					
**Model**	**Mean** **Accuracy ± SD**	**AUC** **(95% CI)**	***p*-value** **for AUC**	**Sensitivity**	**Specificity**	**PPV**	**NPV**	**AUC** **(95% CI)**	***p*-value** **for AUC**	**Sensitivity**	**Specificity**	**PPV**	**NPV**
**Neural Network**	0.82 ± 0.04	0.84 (0.66-1)	0.021	0.73	0.86	0.84	0.76	0.96 (0.92-1)	0.000	0.77	0.94	0.88	0.88
**Random Forest**	0.93 ± 0.02	0.93 (0.83-1)	0.003	1	0.86	0.88	1	0.98 (0.96-1)	0.000	1	0.96	0.94	1
**Gradient Boosting**	0.9 ± 0.03	0.89 (0.77-1)	0.007	1	0.82	0.85	1	0.98 (0.96-1)	0.000	0.98	0.94	0.9	0.99
**Logistic Regression**	0.82 ± 0	0.85 (0.68-1)	0.018	0.91	0.73	0.77	0.89	0.91 (0.86-1)	0.000	0.83	0.81	0.71	0.9

**Table 4:** Comparison of performance before and after improvement of HallMark-32 model. A) Performance indexes of the sample quality-adjusted HallMark-32 Random Forest model. B) Accuracy and AUC after model improvement (i.e. sample quality-adjusted), before improvement (No adjustment), and negative control for improvement.

**A)**	**Sample quality-adjusted HAllMark-32**											
**Model**	**Test set (n = 27)**							**All data (n=133)**				
	**Mean** **accuracy**	**AUC (95% CI)**	**P value for AUC**	**Sensitivity**	**Specificity**	**PPV**	**NPV**	**AUC**	**Sensitivity**	**Specificity**	**PPV**	**NPV**
**Random Forest**	0.95±0.01	0.991 (0.96-1)	0.001	1	0.91	0.92	1	1	1	0.98	0.96	1
**B)**	**HallMark-32 (Test set, n = 27)**											
	**Sample quality-adjusted**			**No adjustment**		**Negative control for improvement**						
**Accuracy**	0.95			0.91		0.84						
**AUC(95% CI)**	0.991 (0.96-1)			0.945 (0.86-1)		0.936 (0.836-1)						
**P value for AUC**	0.001			0.002		0.003						
