# Supplementary material for: Hallmark microRNA signature in liquid biopsy identifies hepatocellular carcinoma and differentiates it from liver metastasis
Source: J Cancer. 2021 Jun 1;12(15):4585–94. doi: 10.7150/jca.59933 (PMC8210546; doi:10.7150/jca.59933)
Supplement: Supplementary file 1 — Supplementary figure and tables. [file jcav12p4585s1.pdf]

Supplementary materials:

Table S1: Primer sequences for HallMark-32

microRNA target	Primer Sequence
**miR-101-3p**	CGCATACAGTACTGTGATAACTGAA
**miR-122-5p**	TGG AGT GTG ACA ATG GTG TTT G
**miR-1246**	GCAATGGATTTTTGGAGCA
**miR-125A-5P**	GAGACCCTTTAACCTGTGAAAAA
**miR-125b-5p**	CTC CCT GAG ACC CTA ACT TGT G
**miR-145-5p**	CAGTTTTCCCAGGAATCCCTAA
**miR-148a-3p**	CGC TCA GTG CAC TAC AGA ACT TT
**miR-150-5p**	TCCCAACCCTTGTACCAGTGAA
**miR-18a-5p**	AAGGTGCATCTAGTGCAGATAGAAA
**miR-191-5p**	AAC GGA ATC CCA AAA GCA G
**miR-192-5p**	CTG ACC TAT GAA TTG ACA GCC AAA
**miR-193a-5p**	GTC TTT GCG GGC GAG AT
**miR-19a-3p**	TGTGCAAATCTATGCAAAACTGA
**miR-21-5p**	AGC TTA TCA GAC TGA TGT TGA AAA A
**miR-214-3p**	GCA CAG CAG GCA CAG ACA G
**miR-22-5p**	AGT TCT TCA GTG GCA AGC TTT A
**miR-221-3p**	GCG AGC TAC ATT GTC TGC TG

**miR-222-3p**	GCT ACA TCT GGC TAC TGG GTA AA
**miR-223-3p**	TGT CAG TTT GTC AAA TAC CCC AAA
**miR-23a-3p**	ACA TTG CCA GGG ATT TCC AA
**miR-26a-5p**	TTC AAG TAA TCC AGG ATA GGC TAA A
**miR-29a-3p**	GCA CCA TCT GAA ATC GGT TAA A
**miR-30c-5p**	GCA GCA TGT AAA CAT CCT ACA CTC T
**miR-320d**	AAAGCTGGGTTGAGAGGAAA
**miR-365a-3p**	CGTAATGCCCCTAAAAATCCTT
**miR-374a-5p**	CGCCTTATAATACAACCTGATAAGTG
**miR-423-3p**	TGAGGCCCCTCAGTAAAAA
**miR-423-5p**	GGGCAGAGAGCGAGACTTTA
**miR-424-5p**	GCAGCAATTCATGTTTTGAA
**miR-451a**	CGA AAA CCG TTA CCA TTA CTG A
**miR-486-5p**	GAGCTGCCCCGAGAAAAA
**miR-574-3p**	CTCATGCACACACCCACAA

Table S2: The reported evidence of HallMark-32 microRNAs in regulating cancer hallmarks

miRNA	Sustaining Proliferative Signaling	Evading Growth Suppressors	Avoiding Immune Destruction	Enabling Replicative Immortality	Tumor Promoting Inflammation	Activating Invasion / Metastasis	Inducing Angiogenesis	Genome Instability	Resisting Cell Death	Deregulating Cellular Energetics
150-5p	[2]					[1]				
574-3p		[52]								
125b-5p	[3]	[4]				[3]			[4]	
191-5p						[5]				
101-3p	[7]					[7]			[6]	[8]
1246	[9]			[48]		[10]			[9]	
21-5p	[11]			[49]		[11]				
23a-3p		[13]	[12]							
145-5p	[14]	[15]				[14]			[16]	
125a-5p						[18]			[17]	
214-3p	[19]	[20]				[19]				
192-5p				[50]					[21]	
320d	[22]									
18a-5p	[23]								[23]	
26a-5p	[24]					[24]			[24]	
193a-5p	[25]								[25]	
365a-3p										
19a-3p	[28]					[27]				
148a-3p				[51]	[29]	[30]	[30]			
222-3p	[31]								[31]	
423-3p		[32]					[33]			
486-5p	[34]					[34]				
22-5p										[53]
223-3p	[35]								[35]	
423-5p		[32]	[36]						[37]	
374a-5p	[38]					[38]		[38]		
221-3p						[40]		[39]		
30c-5p					[54]					
424-5p	[41]	[42]	[44]			[41]	[43]			
122-5p	[45]					[45]				
29a-3p	[46]					[46]				
451a	[47]					[47]				

Table S3: Expression level of miRNAs in HallMark-32 and the *p*-values results in t-test and ANOVA analysis. Notes: * *p*-values ≤ 0.05; ** *p*-values ≤ 0.01; High CTC value means low microRNA expression.

miRNA	Mean			SEM			Fold-change relative to Healthy			HCC vs. CRCLM (p-value)	HCC vs. Healthy (p-value)	HCC vs. CRCLM vs. Healthy (p-value)
	HCC	Healthy	CRCLM	HCC	Healthy	CRCLM	HCC	Healthy	CRCLM			
101-3p	24.25	24.37	23.6	0.24	1.04	0.26	1.09	1	1.71	0.069	0.909	0.319
122-5p	28.75	29.12	28.84	0.26	0.64	0.23	1.29	1	1.21	0.796	0.593	0.783
1246	25.41	25.73	24.01	0.2	0.95	0.31	1.25	1	3.28	0.000**	0.744	0.005**
125a-5p	30.01	30.37	30.1	0.22	0.24	0.16	1.28	1	1.2	0.725	0.277	0.587
125b-5p	31.38	30.73	30.77	0.21	0.46	0.28	0.64	1	0.98	0.082	0.207	0.218
145-5p	32.34	31.32	30.19	0.27	0.69	0.33	0.49	1	2.18	0.000**	0.170	0.000**
148a-3p	27.59	27.1	26.33	0.23	0.59	0.22	0.71	1	1.71	0.000**	0.443	0.003**
150-5p	28.77	27.62	27.66	0.28	0.85	0.26	0.45	1	0.97	0.004**	0.202	0.041*
18a-5p	27.66	27.39	26.9	0.25	0.63	0.24	0.83	1	1.41	0.028*	0.688	0.146
191-5p	27.74	27.05	26.1	0.24	0.9	0.22	0.62	1	1.94	0.000**	0.463	0.001**
192-5p	29.64	29.34	29.15	0.19	0.46	0.18	0.81	1	1.13	0.062	0.541	0.252
193a-5p	33.25	33.24	32.66	0.16	0.37	0.31	0.99	1	1.5	0.093	0.971	0.228
19a-3p	23.42	23.58	22.43	0.25	1.07	0.24	1.12	1	2.21	0.005**	0.884	0.079
21-5p	24.43	24.24	23.19	0.19	0.52	0.21	0.87	1	2.06	0.000**	0.721	0.000**
214-3p	28.58	29.67	28.72	0.18	0.75	0.13	2.13	1	1.94	0.550	0.164	0.047*
22-5p	30.59	30.1	29.36	0.21	0.46	0.21	0.71	1	1.68	0.000**	0.339	0.001**
221-3p	28.15	27.27	26.29	0.21	0.36	0.25	0.55	1	1.97	0.000**	0.042*	0.000**
222-3p	29.65	29.43	28.47	0.21	0.79	0.22	0.86	1	1.95	0.000**	0.788	0.01**
223-3p	25.72	24.14	22.81	0.25	0.73	0.28	0.33	1	2.51	0.000**	0.045*	0.000**
23a-3p	28.04	26.94	25.69	0.21	0.57	0.24	0.47	1	2.38	0.000**	0.077	0.000**
26a-5p	28.14	26.77	26.1	0.26	0.62	0.25	0.39	1	1.6	0.000**	0.046*	0.000**
29a-3p	26.54	26.27	25.19	0.23	0.93	0.24	0.83	1	2.12	0.000**	0.781	0.011**
30c-5p	25.85	25.04	25.05	0.17	0.31	0.17	0.57	1	0.99	0.001**	0.026*	0.004**
320d	33.44	33.61	32.64	0.22	0.31	0.21	1.13	1	1.96	0.01**	0.657	0.009**
365a-3p	28.63	29.2	28.75	0.12	0.2	0.09	1.48	1	1.37	0.474	0.018**	0.027*
374a-5p	28.38	27.51	27	0.29	0.67	0.23	0.55	1	1.43	0.000**	0.240	0.005**
423-3p	29.51	30.35	29.54	0.23	0.23	0.19	1.79	1	1.75	0.917	0.012**	0.062
423-5p	28.69	28.34	27.69	0.2	0.52	0.25	0.79	1	1.57	0.002**	0.542	0.02*
424-5p	28.03	27.85	26.64	0.21	0.87	0.24	0.88	1	2.31	0.000**	0.840	0.004**
451a	17.8	17.91	17.33	0.25	1.01	0.22	1.09	1	1.5	0.161	0.910	0.496
486-5p	24.41	24.17	24.08	0.17	0.42	0.16	0.85	1	1.06	0.168	0.598	0.469

574-3p	31.37	31.09	30.6	0.14	0.26	0.17	0.82	1	1.4	0.001**	0.346	0.004**

Table S4: Data overview for the sample quality data in HCC, Healthy, and CRCLM

Samples quality data	Median			SEM		
	HCC	Healthy	CRCLM	HCC	Healthy	CRCLM
miR-150-5p specificity	1.00	1.00	1.00	0.00	0.05	0.00
miR-547-3p specificity	0.50	1.00	0.50	0.93	0.06	0.04
miR-125b-5p specificity	1.00	1.00	1.00	0.00	0.03	0.02
miR-191-5p specificity	1.00	1.00	1.00	0.00	0.00	0.01
miR-101-3p specificity	1.00	1.00	1.00	0.00	0.05	0.00
miR-1246 specificity	1.00	1.00	1.00	0.00	0.05	0.01
miR-21-5p specificity	1.00	1.00	1.00	0.00	0.05	0.00
miR-23a-3p specificity	1.00	1.00	1.00	0.03	0.05	0.01
miR-145-5p specificity	1.00	1.00	1.00	0.00	0.05	0.00
miR-125a-5p specificity	1.00	1.00	1.00	0.00	0.00	0.00
miR-214-3p specificity	0.25	0.75	0.25	0.05	0.08	0.05
miR-192-5p specificity	1.00	1.00	1.00	0.03	0.06	0.00
miR-320d specificity	1.00	1.00	1.00	0.02	0.00	0.00
miR-18a-5p specificity	1.00	1.00	1.00	0.00	0.00	0.00
miR-26a-5p specificity	1.00	1.00	1.00	0.00	0.00	0.00
miR-193a-5p specificity	0.50	1.00	1.00	0.05	0.08	0.04
miR-365a-3p specificity	1.00	0.50	0.50	0.02	0.05	0.03
miR-19a-3p specificity	1.00	1.00	1.00	0.00	0.05	0.00
miR-148a-3p specificity	1.00	1.00	1.00	0.00	0.00	0.00
miR-222-3p specificity	1.00	1.00	1.00	0.00	0.05	0.02
miR-423-3p specificity	1.00	1.00	1.00	0.02	0.00	0.02
miR-486-5p specificity	1.00	1.00	1.00	0.00	0.00	0.00
miR-22-5p specificity	1.00	1.00	1.00	0.00	0.00	0.00
miR-223-3p specificity	1.00	1.00	1.00	0.00	0.00	0.00
miR-423-5p specificity	1.00	1.00	1.00	0.00	0.00	0.01
miR-374a-5p specificity	1.00	1.00	1.00	0.00	0.00	0.00
miR-221-3p specificity	1.00	1.00	1.00	0.05	0.00	0.04

**miR-30c-5p specificity**	1.00	1.00	1.00	0.03	0.04	0.02
**miR-424-5p specificity**	1.00	1.00	1.00	0.00	0.02	0.00
**miR-122-5p specificity**	1.00	1.00	1.00	0.02	0.03	0.00
**miR-29a-3p specificity**	1.00	1.00	1.00	0.00	0.02	0.00
**miR-451a specificity**	1.00	1.00	1.00	0.00	0.00	0.01
**RNA amount (ug/uL)**	23.00	47.00	51.50	5.57	13.19	8.34
**A260/A280 from RNA**	1.19	1.27	1.12	0.03	0.07	0.03
**A260/A230 from RNA**	0.06	0.09	0.09	0.02	0.04	0.01
**DNA amount (ug/uL)**	1430.55	1533.50	1565.70	24.23	32.10	19.50
**A260/A280 from cDNA**	2.11	2.16	2.13	0.02	0.01	0.01
**A260/A230 from cDNA**	1.79	1.78	1.62	0.04	0.07	0.03

Table S5: Ten cancer hallmarks in HCC and their associated molecular pathways [55]

HCC Hallmarks	Molecular Mechanism of HCC induced by HBV
**Sustaining Proliferative Signaling**	PI3K/Akt, Ras/Raf/MAPK, Src, YAP, Wnt/β-catenin, cyclin D, c-myc, c-jun, EGFR, ccND1, TGF-β, Clusterin, DNMT3b, NOX-4, STAT3
**Evading growth suppressors**	p53, p18
**Avoiding immune destruction**	Immune dysregulation, IFN-β, PDL-1, TGF-β, IL-28
**Enabling replicative immortality**	HBV DNA integration, nTERT
**Tumor promoting inflammation**	Endoplasmic reticulum ROS, NF-Kβ, TNF-α, GSTP1, Mitochondria ROS, IL-6
**Activating invasion and metastasis**	TGF-β, IncRNA-ATB
**Inducing angiogenesis**	VEGF, HIFα, MTA1
**Genome instability and mutation**	Endoplasmic reticulum ROS, HBV DNA integration, Mitochondria ROS, UV-DDBI
**Resisting cell death**	p53, p18 mcl-1, Notch, apoptosis, HBV DNA integration
**Deregulating cellular energetics**	Hypoxia induced lactic acid biosynthesis

Table S6: Probability cut-off values and the associated sensitivity and specificity in ROC curve analysis

	HallMark-32 sample quality-adjusted model (n=133)	
**Probability Cut-off**	**Sensitivity**	**1 - Specificity**
**0.29**	1.00	0.05
**0.39**	1.00	0.04
**0.55**	1.00	0.02
**0.61**	0.98	0.02
**0.63**	0.98	0.01
**0.66**	0.96	0.01
**0.72**	0.94	0.01
**0.78**	0.92	0.01

Figure S1: Bar chart of 32 miRNA CT values in HCC, Healthy and CRCLM groups and fold change relative to healthy. The *p*-values at the top right corner represent the *p*-values in the ANOVA test comparing the CT values in three groups (HCC, Healthy and CRCLM).

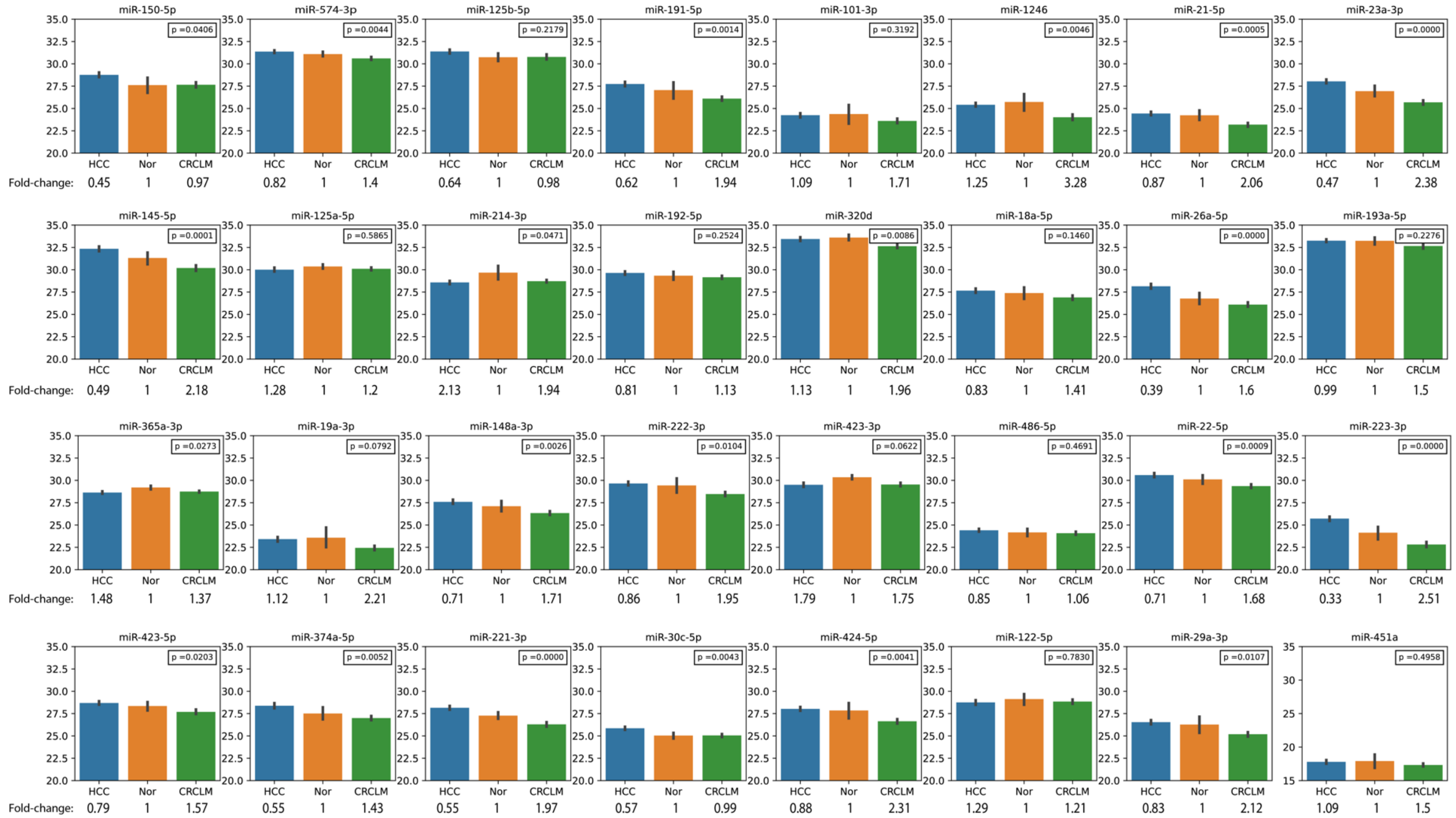

References:

[1] Li T, Xie J, Shen C, Cheng D, Shi Y, Wu Z, et al. (2014) miR-150-5p Inhibits Hepatoma Cell Migration and Invasion by Targeting MMP14. PLoS ONE 9(12): e115577. https://doi.org/10.1371/journal.pone.0115577

[2] Sun W, Zhang Z, Wang J, et al. MicroRNA-150 suppresses cell proliferation and metastasis in hepatocellular carcinoma by inhibiting the GAB1-ERK axis. Oncotarget. 2016;7(10):11595-11608. doi:10.18632/oncotarget.7292

[3] Hua S, Quan Y, Zhan M, Liao H, Li Y, Lu L. miR-125b-5p inhibits cell proliferation, migration, and invasion in hepatocellular carcinoma via targeting TXNRD1. Cancer Cell Int. 2019;19:203. Published 2019 Jul 30. doi:10.1186/s12935-019-0919-6

[4] Wang Y, Qin X, Guo T, Liu P, Wu P, Liu Z. Up-regulation of CDK16 by multiple mechanisms in hepatocellular carcinoma promotes tumor progression [retracted in: J Exp Clin Cancer Res. 2019 May 23;38(1):215]. J Exp Clin Cancer Res. 2017;36(1):97. Published 2017 Jul 17. doi:10.1186/s13046-017-0569-2

[5] Gao Y, Luo T, Ouyang X, Zhu C, Zhu J, Qin X. IGF2BP3 and miR191-5p synergistically increase HCC cell invasiveness by altering ZO-1 expression. Oncol Lett. 2020;20(2):1423-1431. doi:10.3892/ol.2020.11693

[6] Sun W, Zhang Q, Wu Z, Xue N. miR-101-3p sensitizes hepatocellular carcinoma cells to oxaliplatin by inhibiting Beclin-1-mediated autophagy. Int J Clin Exp Pathol. 2019;12(6):2056-2065. Published 2019 Jun 1.

[7] Yan S, Shan X, Chen K, et al. LINC00052/miR-101-3p axis inhibits cell proliferation and metastasis by targeting SOX9 in hepatocellular carcinoma. Gene. 2018;679:138-149. doi:10.1016/j.gene.2018.08.038

[8] Cui G, Wang H, Liu W, et al. Glycogen Phosphorylase B Is Regulated by miR101-3p and Promotes Hepatocellular Carcinoma Tumorigenesis. Front Cell Dev Biol. 2020;8:566494. Published 2020 Nov 25. doi:10.3389/fcell.2020.566494

[9] Zhang Q, Cao LY, Cheng SJ, Zhang AM, Jin XS, Li Y. p53-induced microRNA-1246 inhibits the cell growth of human hepatocellular carcinoma cells by targeting NFIB. Oncol Rep. 2015;33(3):1335-1341. doi:10.3892/or.2015.3715

[10] Sun Z, Meng C, Wang S, et al. MicroRNA-1246 enhances migration and invasion through CADM1 in hepatocellular carcinoma. BMC Cancer. 2014;14:616. Published 2014 Aug 27. doi:10.1186/1471-2407-14-616

[11] Cai M, Shao W, Yu H, Hong Y, Shi L. Paeonol Inhibits Cell Proliferation, Migration and Invasion and Induces Apoptosis in Hepatocellular Carcinoma by Regulating miR-21-5p/KLF6 Axis. Cancer Manag Res. 2020;12:5931-5943. Published 2020 Jul 17. doi:10.2147/CMAR.S254485

[12] Liu J, Fan L, Yu H, et al. Endoplasmic Reticulum Stress Causes Liver Cancer Cells to Release Exosomal miR-23a-3p and Up-regulate Programmed Death Ligand 1 Expression in Macrophages. Hepatology. 2019;70(1):241-258. doi:10.1002/hep.30607

[13] Xiang Y, Yang Y, Lin C, Wu J, Zhang X. MiR-23a-3p promoted G1/S cell cycle transition by targeting protocadherin17 in hepatocellular carcinoma. J Physiol Biochem. 2020;76(1):123-134. doi:10.1007/s13105-020-00726-4

[14] Liang H, Sun H, Yang J, Yi C. miR-145-5p reduces proliferation and migration of hepatocellular carcinoma by targeting KLF5. Mol Med Rep. 2018;17(6):8332-8338. doi:10.3892/mmr.2018.8880

[15] Dong G, Zhang S, Shen S, et al. SPATS2, negatively regulated by miR-145-5p, promotes hepatocellular carcinoma progression through regulating cell cycle. Cell Death Dis. 2020;11(10):837. Published 2020 Oct 9. doi:10.1038/s41419-020-03039-y
[16] Lupini L, Pepe F, Ferracin M, et al. Over-expression of the miR-483-3p overcomes the miR-145/TP53 pro-apoptotic loop in hepatocellular carcinoma. Oncotarget. 2016;7(21):31361-31371. doi:10.18632/oncotarget.8913
[17] Ming M, Ying M, Ling M. miRNA-125a-5p inhibits hepatocellular carcinoma cell proliferation and induces apoptosis by targeting TP53 regulated inhibitor of apoptosis 1 and Bcl-2-like-2 protein. Exp Ther Med. 2019;18(2):1196-1202. doi:10.3892/etm.2019.7674
[18] Tang H, Li RP, Liang P, Zhou YL, Wang GW. miR-125a inhibits the migration and invasion of liver cancer cells via suppression of the PI3K/AKT/mTOR signaling pathway. Oncol Lett. 2015;10(2):681-686. doi:10.3892/ol.2015.3264
[19] Liu C, Shang Z, Ma Y, Ma J, Song J. HOTAIR/miR-214-3p/FLOT1 axis plays an essential role in the proliferation, migration, and invasion of hepatocellular carcinoma. Int J Clin Exp Pathol. 2019;12(1):50-63. Published 2019 Jan 1.
[20] Li Y, Li Y, Chen Y, et al. MicroRNA-214-3p inhibits proliferation and cell cycle progression by targeting MELK in hepatocellular carcinoma and correlates cancer prognosis [published correction appears in Cancer Cell Int. 2018 Apr 10;18:55]. Cancer Cell Int. 2017;17:102. Published 2017 Nov 7. doi:10.1186/s12935-017-0471-1
[21] Nielsen KO, Jacobsen KS, Mirza AH, et al. Hepatitis B virus upregulates host microRNAs that target apoptosis-regulatory genes in an in vitro cell model. Exp Cell Res. 2018;371(1):92-103. doi:10.1016/j.yexcr.2018.07.044
[22] Li W, Ding X, Wang S, et al. Downregulation of serum exosomal miR-320d predicts poor prognosis in hepatocellular carcinoma. J Clin Lab Anal. 2020;34(6):e23239. doi:10.1002/jcla.23239
[23] Chen Z, Ma Y, Pan Y, et al. Long Noncoding RNA RP5-833A20.1 Suppresses Tumorigenesis In Hepatocellular Carcinoma Through Akt/ERK Pathway By Targeting miR-18a-5p. Onco Targets Ther. 2019;12:10717-10726. Published 2019 Dec 6. doi:10.2147/OTT.S219797
[24] Li ML, Zhang Y, Ma LT. LncRNA HCG11 accelerates the progression of hepatocellular carcinoma via miR-26a-5p/ATG12 axis. Eur Rev Med Pharmacol Sci. 2019;23(24):10708-10720. doi:10.26355/eurrev_201912_19771
[25] Wang JT, Wang ZH. Role of miR-193a-5p in the proliferation and apoptosis of hepatocellular carcinoma. Eur Rev Med Pharmacol Sci. 2018;22(21):7233-7239. doi:10.26355/eurrev_201811_16257
[26] Jin Y, Wong YS, Goh BKP, et al. Circulating microRNAs as Potential Diagnostic and Prognostic Biomarkers in Hepatocellular Carcinoma. Sci Rep. 2019;9(1):10464. Published 2019 Jul 18. doi:10.1038/s41598-019-46872-8
[27] Jiang XM, Yu XN, Liu TT, et al. microRNA-19a-3p promotes tumor metastasis and chemoresistance through the PTEN/Akt pathway in hepatocellular carcinoma. Biomed Pharmacother. 2018;105:1147-1154. doi:10.1016/j.biopha.2018.06.097
[28] Sun HX, Yang ZF, Tang WG, et al. MicroRNA-19a-3p regulates cell growth through modulation of the PIK3IP1-AKT pathway in hepatocellular carcinoma. J Cancer. 2020;11(9):2476-2484. Published 2020 Feb 10. doi:10.7150/jca.37748
[29] Deng Y, Wang J, Huang M, Xu G, Wei W, Qin H. Inhibition of miR-148a-3p resists hepatocellular carcinoma progress of hepatitis C virus infection through suppressing c-Jun and MAPK pathway. J Cell Mol Med. 2019;23(2):1415-1426. doi:10.1111/jcmm.14045
[30] Song SK, Jung WY, Park SK, Chung CW, Park Y. Significantly different expression levels of microRNAs associated with vascular invasion in hepatocellular carcinoma and their prognostic significance after surgical resection. PLoS One. 2019;14(9):e0216847. Published 2019 Sep 12. doi:10.1371/journal.pone.0216847

[31] Wang X, Cheng ML, Gong Y, Ma WJ, Li B, Jiang YZ. LncRNA DANCR promotes ATG7 expression to accelerate hepatocellular carcinoma cell proliferation and autophagy by sponging miR-222-3p. Eur Rev Med Pharmacol Sci. 2020;24(17):8778-8787. doi:10.26355/eurrev_202009_22816
[32] Lin J, Huang S, Wu S, et al. MicroRNA-423 promotes cell growth and regulates G(1)/S transition by targeting p21Cip1/Waf1 in hepatocellular carcinoma. Carcinogenesis. 2011;32(11):1641-1647. doi:10.1093/carcin/bgr199
[33] de Oliveira ARCP, Castanhole-Nunes MMU, Biselli-Chicote PM, et al. Differential expression of angiogenesis-related miRNAs and VEGFA in cirrhosis and hepatocellular carcinoma. Arch Med Sci. 2020;16(5):1150-1157. Published 2020 Aug 10. doi:10.5114/aoms.2020.97967
[34] He J, Xiao B, Li X, He Y, Li L, Sun Z. MiR-486-5p Suppresses Proliferation and Migration of Hepatocellular Carcinoma Cells through Downregulation of the E3 Ubiquitin Ligase CBL. Biomed Res Int. 2019;2019:2732057. Published 2019 Dec 28. doi:10.1155/2019/2732057
[35] Wan L, Yuan X, Liu M, Xue B. miRNA-223-3p regulates NLRP3 to promote apoptosis and inhibit proliferation of hep3B cells. Exp Ther Med. 2018;15(3):2429-2435. doi:10.3892/etm.2017.5667
[36] Yu Z, Zhao H, Feng X, et al. Long Non-coding RNA FENDRR Acts as a miR-423-5p Sponge to Suppress the Treg-Mediated Immune Escape of Hepatocellular Carcinoma Cells. Mol Ther Nucleic Acids. 2019;17:516-529. doi:10.1016/j.omtn.2019.05.027
[37] Stiuso P, Potenza N, Lombardi A, et al. MicroRNA-423-5p Promotes Autophagy in Cancer Cells and Is Increased in Serum From Hepatocarcinoma Patients Treated With Sorafenib. Mol Ther Nucleic Acids. 2015;4:e233. Published 2015 Mar 17. doi:10.1038/mtna.2015.8
[38] Lin Q, Zhou CR, Bai MJ, et al. Exosome-mediated miRNA delivery promotes liver cancer EMT and metastasis. Am J Transl Res. 2020;12(3):1080-1095. Published 2020 Mar 15.
[39] Chen Z, Xiang B, Qi L, Zhu S, Li L. miR-221-3p promotes hepatocellular carcinogenesis by downregulating O6-methylguanine-DNA methyltransferase. Cancer Biol Ther. 2020;21(10):915-926. doi:10.1080/15384047.2020.1806642
[40] Yang J, Cui R, Liu Y. MicroRNA-212-3p inhibits paclitaxel resistance through regulating epithelial-mesenchymal transition, migration and invasion by targeting ZEB2 in human hepatocellular carcinoma. Oncol Lett. 2020;20(4):23. doi:10.3892/ol.2020.11884
[41] Du H, Xu Q, Xiao S, et al. MicroRNA-424-5p acts as a potential biomarker and inhibits proliferation and invasion in hepatocellular carcinoma by targeting TRIM29. Life Sci. 2019;224:1-11. doi:10.1016/j.lfs.2019.03.028
[42] Li D, Tang X, Li M, Zheng Y. Long noncoding RNA DLX6-AS1 promotes liver cancer by increasing the expression of WEE1 via targeting miR-424-5p. J Cell Biochem. 2019;120(8):12290-12299. doi:10.1002/jcb.28493
[43] Teng F, Zhang JX, Chang QM, et al. LncRNA MYLK-AS1 facilitates tumor progression and angiogenesis by targeting miR-424-5p/E2F7 axis and activating VEGFR-2 signaling pathway in hepatocellular carcinoma [published correction appears in J Exp Clin Cancer Res. 2020 Dec 9;39(1):277]. J Exp Clin Cancer Res. 2020;39(1):235. Published 2020 Nov 9. doi:10.1186/s13046-020-01739-z
[44] Yang Z, Zi Q, Xu K, Wang C, Chi Q. Development of a macrophages-related 4-gene signature and nomogram for the overall survival prediction of hepatocellular carcinoma based on WGCNA and LASSO algorithm. Int Immunopharmacol. 2021;90:107238. doi:10.1016/j.intimp.2020.107238

[45] Gao J, Yin X, Yu X, Dai C, Zhou F. Long noncoding LINC01551 promotes hepatocellular carcinoma cell proliferation, migration, and invasion by acting as a competing endogenous RNA of microRNA-122-5p to regulate ADAM10 expression. J Cell Biochem. 2019;120(10):16393-16407. doi:10.1002/jcb.28549
[46] Xiao Z, Wang Y, Ding H. XPD suppresses cell proliferation and migration via miR-29a-3p-Mdm2/PDGF-B axis in HCC. Cell Biosci. 2019;9:6. Published 2019 Jan 5. doi:10.1186/s13578-018-0269-4
[47] Wei GY, Hu M, Zhao L, Guo WS. MiR-451a suppresses cell proliferation, metastasis and EMT via targeting YWHAZ in hepatocellular carcinoma. Eur Rev Med Pharmacol Sci. 2019;23(12):5158-5167. doi:10.26355/eurrev_201906_18180
[48] Chai S, Ng KY, Tong M, et al. Octamer 4/microRNA-1246 signaling axis drives Wnt/β-catenin activation in liver cancer stem cells. Hepatology. 2016;64(6):2062-2076. doi:10.1002/hep.28821
[49] Wang S, Cai L, Zhang F, Shang X, Xiao R, Zhou H. Inhibition of EZH2 Attenuates Sorafenib Resistance by Targeting NOTCH1 Activation-Dependent Liver Cancer Stem Cells via NOTCH1-Related MicroRNAs in Hepatocellular Carcinoma. Transl Oncol. 2020;13(3):100741.
[50] Gu Y, Wei X, Sun Y, et al. miR-192-5p Silencing by Genetic Aberrations Is a Key Event in Hepatocellular Carcinomas with Cancer Stem Cell Features. Cancer Res. 2019;79(5):941-953. doi:10.1158/0008-5472.CAN-18-1675
[51] Li X, Wang L, Cao X, et al. Casticin inhibits stemness of hepatocellular carcinoma cells via disrupting the reciprocal negative regulation between DNMT1 and miR-148a-3p. Toxicol Appl Pharmacol. 2020;396:114998. doi:10.1016/j.taap.2020.114998
[52] Zha, Zhongming & Jia, Fuxin & Hu, Pingan & Mai, Erhui & Lei, Ting. (2020). MicroRNA-574-3p inhibits the malignant behavior of liver cancer cells by targeting ADAM28. Oncology Letters. 20. 10.3892/ol.2020.11852.
[53] Luo, LJ., Zhang, LP., Duan, CY. et al. The inhibition role of miR-22 in hepatocellular carcinoma cell migration and invasion via targeting CD147. Cancer Cell Int 17, 17 (2017). https://doi.org/10.1186/s12935-016-0380-8
[54] Budhu A, Jia HL, Forgues M, et al. Identification of metastasis-related microRNAs in hepatocellular carcinoma. Hepatology. 2008;47(3):897-907. doi:10.1002/hep.22160
[55] D'Souza, S., et al., Molecular mechanisms of viral hepatitis induced hepatocellular carcinoma. World J Gastroenterol, 2020. 26(38): p. 5759-5783.
